# Isolation, characterization, and complete genome analysis of P1312, a thermostable bacteriophage that infects *Thermobifida fusca*

**DOI:** 10.3389/fmicb.2015.00959

**Published:** 2015-09-15

**Authors:** Jatuporn Cheepudom, Cheng-Cheng Lee, Bingfu Cai, Menghsiao Meng

**Affiliations:** Graduate Institute of Biotechnology, National Chung Hsing UniversityTaichung, Taiwan

**Keywords:** bacteriophage, *Thermobifida fusca*, lignocellulosic agricultural wastes, large terminase subunit, amidase, phage purification, endolysins, actinobacteriophage

## Abstract

*Thermobifida fusca* is a moderately thermophilic and cellulolytic actinobacterium. It is of particular interest due to its ability to not only produce a variety of biotechnologically relevant enzymes but also serve as an alternative host for metabolic engineering for the production of valuable chemicals from lignocellulosic agricultural wastes. No bacteriophage that infects *T. fusca* has been reported, despite its potential impacts on the utilization of *T. fusca*. In this study, an extremely thermostable bacteriophage P1312 that infects *T. fusca* was isolated from manure compost. Electron microscopy showed that P1312 has an icosahedral head and a long flexible non-contractile tail, a characteristic of the family *Siphoviridae*. P1312 has a double-stranded DNA genome of 60,284 bp with 93 potential ORFs. Thirty-one ORFs encode proteins having putative biological functions. The genes involved in phage particle formation cluster together in a region of approximately 16 kb, followed by a segment containing genes presumably for DNA degradation/modification and cell wall disruption. The genes required for DNA replication and transcriptional control are dispersed within the rest of the genome. Phylogenetic analysis of large terminase subunit suggests that P1312 is a headful packaging phage containing a chromosome with circularly permuted direct terminal repeats.

## Introduction

*Thermobifida fusca*, a moderately thermophilic soil actinobacterium, is known for its ability to produce a battery of cellulolytic enzymes (Maki et al., 2009; Adav et al., 2010; Gomez del Pulgar and Saadeddin, 2014). Sequence analyses of the genome of *T. fusca* YX strain suggests that it produces nine cellulases (including endocellulase, exocellulase, and cellobiosidase), at least five hemicellulose hydrolysis-related enzymes and many other glycoside hydrolases (Lykidis et al., 2007). Recent studies demonstrated that *T. fusca* may also secret lignin degradation-promoting enzymes. For example, a copper-containing polyphenol oxidase exhibits an activity for oxidation of phenolic lignin related compounds, and this activity boosts the digestion function of cellulase/xylanase toward sugarcane bagasse (Chen et al., 2013). Besides cellulolytic enzymes, *T. fusca* also produces a variety of oxidoreductases, such as heme-containing peroxidase (van Blooise et al., 2010) and catalase (Lončar and Fraaije, 2015), which are potentially useful in industries for detoxification and decolorization.

Cellulosic biomass is a low cost, abundant and renewable source for biofuels. To utilize it, great efforts have been devoted to engineer *Saccharomyces cerevisiae* so that the genetically modified yeasts can ferment xylose, cellobiose, and cello-oligosaccharides for bioethanol production (Katahira et al., 2006; Ha et al., 2011). Nonetheless, directly using the recalcitrant lignocellulose for biofuel production is still a challenging task. *T. fusca* has potential to be an alternative host for metabolic engineering to transform the sugars embedded in lignocellulose into biofuels and green chemicals. For example, an engineered *T. fusca* strain was able to convert untreated plant biomass to 1-propanol after an exogenous gene of bifunctional butyraldehyde/alcohol dehydrogenase was inserted into its genome (Deng and Fong, 2011). Despite this encouraging success, more molecular biology tools such as expression vectors and efficient transformation methods need to be developed before *T. fusca* can be fully modified for the purpose of producing valuable commodities from various cellulosic agricultural wastes.

Bacteriophages have contributed to the development of a variety of molecular tools for biotechnology. For example, the origin of replication from f1 and λ phages was used in the construction of plasmids for single-stranded DNA production (Reece, 2004) and routine recombinant DNA operation, respectively (Boyd and Sherratt, 1995). In addition, recombinases from bacteriophages such as phage λ, P1, and PY54 have been widely used to modify prokaryotic species and to create transgenic animals and plants (Sauer and Henderson, 1988; Nafissi and Slavcev, 2014). On the other hand, bacteriophages have potentials to foul industries that use bacteria to produce fermented products or bioactive molecules. No detailed reports of bacteriophages that infect *T. fusca* are present in the literature. Thus, we set out to isolate *T. fusca*-infecting phages with long-term goals to understand the bacterium-phage interactions and look for useful genetic elements for the development of molecular tools specific for *T. fusca*. A thermostable tailed bacteriophage of the *Siphoviridae* family was then isolated in this study.

Tailed bacteriophages constitute the *Caudovirales* order. Phages with contractile tails are further subdivided into the *Myoviridae*, while those with short and long non-contractile tails are into *Podoviridae* and *Siphoviridae*, respectively. Tails are critical to the infection because they contain proteins required for the specific recognition of the hosts and trigger DNA release from the heads. The genomes of tailed bacteriophages are composed of several functional modules. For *Siphoviridae*, the modular arrangement as follows: packaging, head morphogenesis, tail morphogenesis, lysis, recombination, lytic/lysogenic control, excision, and DNA replication modules is seen repeatedly in numerous temperate phages of Gram-positive low GC-content bacteria (Stevens et al., 2011), whereas lambdoid phages that infect Gram-negative bacteria have a genome consisting of modules in order of packaging, head morphogenesis, tail morphogenesis, recombination, lytic/lysogenic control, DNA replication, and lysis (Campbell, 1994). Characteristics and genome analysis of this newly discovered *T. fusca*-infecting phage are addressed herein.

## Materials and methods

### Bacterial strain

*T. fusca* NTU22 strain (Chen et al., 2013) was routinely cultivated by transferring 10^7^ spores into 50 ml CYC medium (30 g sucrose, 6 g Casamino acids, 3 g NaNO_3_, 2 g yeast extract, 1 g K_2_HPO_4_, 0.5 g KCl, 0.5 g MgSO_4_·7H_2_O, 0.01 g FeSO_4_·7H_2_O, 1 l distilled water, pH 8.0) and incubated aerobically at 50°C with 200 rpm shaking.

### Phage isolation

Compost collected from eight different sites in the suburbs of Taichung city, Taiwan, was the source of phage screening. One gram of the compost was added into 10 ml Luria-Bertani medium (LB) and incubated aerobically at 50°C overnight. One ml aliquot of the sample was irradiated (120 mJ/cm^2^, 30 s) using a CL-1000 UV crosslinker (UVP, California, USA) before being mixed with 0.1 ml *T. fusca* spore (10^8^ cfu/ml) and 15 ml CYC media with 0.7% agar. The warm mixture was immediately poured onto a solidified CYC agar plate and incubated at 50°C for 2 days. One of the plaques formed in the bacterial lawn was transferred into 50 ml 1-day-old culture of *T. fusca* and further incubated at 50°C for 1 day. The bacterial lysate was centrifuged, and the supernatant was sterilized using a 0.45-μm filter and stored at 4°C. The isolated bacteriophage capable of infecting *T. fusca* was named P1312 hereafter.

### Phage titer

Phage titer was estimated by plaque assay method. Briefly, 1 ml phage sample at the 10^7^–10^9^ dilution was mixed with 10^7^ spore of *T. fusca* in 15 ml pre-warmed (50°C) CYC soft agar medium, and the mixture was poured evenly onto a CYC solid agar medium. The solidified plates were then incubated at 50°C for 2 days, and the number of plaques formed in the lawn of *T. fusca* was counted to determine the phage titer.

### Phage stability, adsorption, and burst size

To determine the structural stability of P1312, the purified phage sample was diluted in water and incubated at temperatures ranging from 60–95°C for the indicated periods of time (10–45 min), and the residual infectivity was determined by the plaque assay. The burst size of P1312 was determined by one-step growth method (Lin et al., 2012) with slight modifications. Briefly, P1312 was added into an overnight culture of *T. fusca* in 20 mM phosphate buffer (pH 8.0) to give a multiplicity of infection (MOI) of 1.0 according to the initial spore number of *T. fusca*. The mixture was incubated at room temperature for 10 min to allow the phage particles to attach to the host. The supernatant, after centrifugation at 12,000 rpm for 5 min, was collected to determine the number of unattached phage particles. The percentage of adsorption was calculated using the formula [(initial titer-titer in supernatant)/initial titer × 100%]. The pelleted cells were washed two more times with CYC medium and resuspended in 50 ml of pre-warmed CYC and incubated at 50°C. Aliquots of 1 ml culture were taken at intervals and the phage titers in the clarified supernatant were then determined as aforementioned. The burst size (B_s_) of P1312 was calculated as B_s_ = P_t_/P_o_ where P_t_ is the phage titer at the plateau phase and P_o_ is the initial infective titer, which was estimated on the basis of the plaque-forming units arising from the initially washed cells.

### Phage purification

Hundred milliliter broth of *T. fusca*, which had been cultivated for 1 day, was inoculated with 1 ml of P1312 stock (10^9^ pfu/ml) and the cultivation was continued for another 2 days at 50°C, 200 rpm. The clarified broth, after centrifugation at 12,000 rpm, 4°C, for 10 min, was adjusted to contain 1.5 M NaCl and 10% (w/v) polyethylene glycol (PEG) 8000. The mixture was placed on ice for at least 1 h and subjected to centrifugation at 12,000 rpm, 4°C, for 15 min to precipitate the phage particles. The pellet was resuspended in TBS buffer (50 mM Tris-HCl [pH 8.0], 150 mM NaCl) that additionally contained 0.25 U/ml DNase I and 10 μg/ml RNase A (Takara Bio). After incubation at 30°C for 1 h, the supernatant was passed through a filter with 0.45 μm pore size and loaded onto a Tricorn 10/600 column packed with Sephacryl S-500HR (Amersham Biosciences). The chromatography was performed with TBS buffer at a flow rate of 0.7 ml/min.

### Transmission electron microscopy (TEM)

The purified phage particles on a carbon-coated copper grid were negatively stained with 2% uranyl acetate (pH 4.0) for 10 min and observed at an accelerating voltage of 120 kV under a transmission electron microscope (Jeol LEM-1400).

### Genome analysis of phage P1312

Genomic DNA was obtained from the purified phage P1312 after phenol/chloroform extraction and ethanol precipitation. Whole genome sequencing of P1312 was performed using Illumina Miseq (Tri-I Biotech, Inc). The count of reads was 115,622 with the average length of 175 bases per read. The sequence data could be assembled into a circular single contig of 60,284 base pairs using the *de novo* assembly algorithm of CLC Genomics Workbench (Qiagen). The nucleotide sequence of the genome of P1312 is deposited at GenBank under accession number KT021004. Identification of potential open reading frames (ORFs) within the phage genome was performed by using Bacterial Annotation System (van Domselaar et al., 2005) as well as Glimmer/RBSfinder program (Delcher et al., 1999), and the functions of proteins encode by the ORFs were predicted based on BLASTp program and conserved domain search (http://www.ncbi.nlm.nih.gov/). The probable replication origin of P1312 was predicted by the GenSkew program (http://genskew.csb.univie.ac.at/).

### Phylogenetic analysis

Protein sequences of terminase large subunit from a variety of bacteriophages were retrieved from the biological database, National Center for Biotechnology Information (NCBI). Multiple sequence alignments of those terminase sequences were performed by using the ClustalW program with default parameters in MEGA 6.0 version (Tamura et al., 2013). Phylogenetic tree was built by the neighbor-joining method and phylogenies were determined by bootstrap analysis of 10,000 replicates in MEGA 6.0 version.

### Identification of proteins associated with P1312 virions

Proteins of P1312, purified via PEG precipitation and gel filtration chromatography, were identified by tandem mass spectrometry using an Applied Biosystems QStar LC-MS/MS spectrometer (Life Technologies Corp., Carlsbad, USA). The obtained spectrometry information was analyzed with Mascot software (Matrix Science Ltd., London, UK) using the NCBI non-redundant database and the specific database created in this study based on the predicted ORFs of phage P1312 (Table 1). The important parameter settings for Mascot analysis were as follows: mass values, monoisotopic; protein mass, unrestricted; peptide mass tolerance, ±0.5 Dalton; fragment mass tolerance, ±0.5 Dalton; and maximal missed cleavages, 2.

**Table 1:** **Predicted open reading frames (ORFs) of P1312 and predicted database matches**.

**ORF**	**Start**	**End**	**Protein kDa (Start)^a^**	**Predicted functions**	**BLASTP^b^(best match)**	**Ident (%)^c^**	**LC-M/M^d^**
001	243	980	27.8 (V)	Phage head morphogenesis protein	WP_017602198/	132/249	38
					*Nocardiopsis lucentensis*	(53)	
002	987	1475	18.3 (V)	Hypothetical protein	WP_030728966	80/131	
					*Streptomyces*	(61)	
003	1462	2805	50.6 (M)	Phage terminase large subunit	WP_027740749	289/435	38
					*Streptomyces*	(66)	
004	5101	2795	83.4 (V)	Phage portal protein	WP_040692131	299/405	14
					*Nocardiopsis lucentensis*	(74)	
				gp23	YP_003714730	36/148	
					phiSASD1 phage	(24)	
005	4301	5080	29.0 (L)	Hypothetical protein	WP_040692134	45/61	
					*Nocardiopsis lucentensis*	(74)	
006	5774	5085	25.2 (V)		No match		44
007	5121	6005	31.3 (V)	Capsid	WP_017602203	225/283	18
					*Nocardiopsis lucentensis*	(80)	
				gp27	YP_003714734	28/91	
					phiSASD1 phage	(31)	
008	6036	6461	15.5 (V)	Phage protein	WP_017602204	84/138	63
					*Nocardiopsis lucentensis*	(61)	
				gp27	YP_003714736	21/47	
					phiSASD1 phage	(45)	
009	6644	6363	10.1 (V)		No match		
010	6954	6376	21.7 (V)		No match		63
011	6487	6963	18.2 (M)	Hypothetical protein	WP_040692137	34/68	
					*Nocardiopsis lucentensis*	(50)	
012	6978	7340	13.3 (M)	Phage head-tail adaptor	WP_017602206	80/120	66
					*Nocardiopsis lucentensis*	(67)	
				gp30	YP_003714737	38/94	
					phiSASD1 phage	(40)	
013	7340	7732	14.8 (V)	Phage tail protein	WP_040692140	49/70	
					*Nocardiopsis lucentensis*	(70)	
				gp32	YP_003714739	43/105	
					phiSASD1 phage	(41)	
014	8508	7699	29.7 (V)		No match		36
015	7720	8157	16.2 (V)	Phage tail protein	WP_026128262	84/130	
					*Nocardiopsis lucentensis*	(65)	
				gp33	YP_003714740	44/124	
					phiSASD1 phage	(35)	
016	8512	8703	7.1 (V)	Phage tail protein	WP_017602209	45/62	
					*Nocardiopsis lucentensis*	(73)	
				gp34	YP_003714741	19/53	
					phiSASD1 phage	(36)	
017	8703	9098	14.3 (M)	Hypothetical protein	WP_040692145	67/116	
					*Nocardiopsis lucentensis*	(58)	
				gp35	YP_003714743	40/116	
					phiSASD1 phage	(34)	
018	9119	9406	11.2 (V)	Phage tail protein	WP_017602211	48/99	
					*Nocardiopsis lucentensis*	(48)	
019	9411	9587	5.9 (V)		No match		
020	9418	14391	177.1 (V)	Tail tape measure protein	WP_040703000	156/362	27
					*Nocardiopsis salina*	(43)	
				gp37	YP_003714744	19/56	
					phiSASD1 phage	(34)	
021	14388	16361	71.7 (V)	Phage tail protein	WP_017602213	356/672	
					*Nocardiopsis lucentensis*	(53)	
				gp38	YP_003714745	47/88	
					phiSASD1 phage	(53)	
022	16358	19648	120.6 (M)	Phosphodiesterase	WP_026128263	520/933	
					*Nocardiopsis lucentensis*	(56)	
				gp38	YP_003714745	126/465	
					phiSASD1 phage	(27)	
023	19670	20212	19.3 (M)	Hypothetical protein	WP_040692409	71/180	23
					*Nocardiopsis lucentensis*	(39)	
024	20237	20497	9.6 (M)		No match		
025	20494	21270	28.4 (M)	O-methyltransferase	YP_024820	63/175	18
					*Actinoplanes* phage phiAsp2	(36)	
026	21359	21267	3.2 (M)		No match		
027	21428	22423	37.2 (V)	Glycosyl/glycerophosphate transferases	WP_017602346	147/213	
					*Nocardiopsis lucentensis*	(69)	
028	22494	23063	20.1 (L)	Hypothetical protein	WP_045936534	35/120	
					*Streptomyces* sp. NRRL S-104	(29)	
029	23346	23068	10.5 (M)	Hypothetical protein	WP_017602347	43/86	
					*Nocardiopsis lucentensis*	(50)	
030	23167	23517	11.9 (V)		No match		
031	23530	23408	4.9 (M)		No match		
032	23850	23530	12.0 (M)		No match		
033	23822	24076	8.5 (V)		No match		
034	24019	23870	5.9 (M)		No match		
035	24099	24554	13.5 (L)		No match		
036	24608	24126	17.6 (L)	Hypothetical protein	WP_031096358	58/114	
					*Streptomyces*	(51)	
037	24451	24597	5.4 (V)		No match		
038	24655	24882	8.6 (M)	Transcriptional regulator	WP_017946204	53/68	
					*Streptomyces* sp. CNS615	(78)	
039	25042	25404	13.5 (V)	Protein involved in exopolysaccharide biosynthesis	WP_042170120	41/124	
					*Streptomyces* sp. NBRC 110035	(33)	
040	25530	25973	16.3 (L)	Sensory protein kinase CreC	WP_043471037	47/109	
					*Kitasatospora* sp. MBT66	(43)	
041	25970	26845	30.8 (M)	N-acetylmuramoyl-L-alanine amidase/Lysin	WP_017607061, *Nocardiopsis*	173/266	34
					*xinjiangensis*	(65)	
042	27063	27344	9.9 (L)	Hypothetical protein (Similar to Tfu_2915)	WP_011293338	45/73	
					*Thermobifida fusca*	(62)	
043	27313	28419	38.9 (L)		No match		
044	28350	27490	32.0 (V)	Transcriptional regulator (Helix-turn-helix family protein)	WP_018223844	54/131	
					*Salinispora pacifica*	(41)	
045	28819	28406	14.5 (M)		No match		
046	29336	28905	16.5 (M)		No match		
047	30751	29333	50.3 (V)	Hypothetical protein	ADJ28745	88/289	
					*Nitrosococcus watsonii* C-113	(30)	
048	29466	29365	3.7 (L)		No match		
049	29438	30544	42.5 (M)	Transposase, IS605 orfB	WP_016188297	364/368	29
					*Thermobifida fusca*	(99)	
050	30724	31998	44.1 (L)		No match		27
051	31767	30778	34.9 (M)	Hypothetical protein	WP_037974524	40/107	14
					*Synergistes jonesii*	(37)	
052	33166	31952	45.3 (M)	Transcriptional regulator	WP_017615773	223/399	
				(Helix-turn-helix XRE-family like proteins)	*Nocardiopsis salina*	(56)	
053	34868	33456	50.7 (V)	Replicative DNA helicase	WP_035111511	174/451	31
					*Corynebacterium freiburgense*	(39)	
054	34798	35892	37.3 (V)		No match		
055	36066	34912	42.3 (V)	Hypothetical protein	WP_040793245	49/130	
					*Nocardia paucivorans*	(38)	
056	37130	36525	22.0 (M)	Hypothetical protein	WP_040271854	37/87	
					*Streptomonospora alba*	(43)	
057	36735	37016	9.8 (V)		No match		
058	37102	38487	45.7 (V)		No match		
059	37779	37183	22.1 (M)		No match		
060	38460	37840	23.7 (M)	Hypothetical protein	WP_017541564	59/168	
					*Nocardiopsis halophila*	(35)	
061	38523	40232	64.3 (L)		No match		
062	40349	38715	55.7 (V)	Hypothetical protein	WP_013475335	89/160	
					*Micromonospora* sp. L5	(56)	
063	42526	40484	72.8 (V)	DNA segregation ATPase FtsK/SpoIII	WP_017602285	253/481	
					*Nocardiopsis lucentensis*	(53)	
064	44174	42486	58.0 (V)	Hypothetical protein	WP_017602419	96/293	
					*Nocardiopsis lucentensis*	(33)	
065	43517	43747	8.7 (M)		No match		
066	44513	44346	6.2 (V)		No match		
067	45035	44577	16.5 (V)	ABC-ATPase	WP_037918697	51/119	
					*Streptomyces yeochonensis*	(43)	
068	45020	46795	67.3 (L)	Intergrase/Recombinase	YP_005087274	155/501	
					*Rhodococcus* phage REQ1	(31)	
069	47265	46846	15.3 (M)		No match		
070	47870	47286	22.4 (V)	Hypothetical protein	WP_036322164	44/113	51
					*Microbispora* sp. ATCC PTA-5024	(39)	
071	47971	48198	8.6 (M)	Transcriptional regulator	WP_012032242	33/63	
				(Helix-turn-helix XRE-family like proteins)	*Pelotomaculum thermopropionicum*	(52)	
072	48520	48948	16.5 (M)		No match		
073	49402	48866	18.2 (L)		No match		
074	49094	49405	11.2 (V)		No match		
075	49402	50229	30.7 (M)		No match		29
076	49935	49747	6.6 (M)		No match		79
077	50446	51360	33.9 (V)	Exodeoxyribonuclease VIII	WP_017972488	156/282	
					*Actinopolyspora halophila*	(55)	
078	51357	52118	28.2 (V)	Hypothetical protein	WP_045740910	117/172	
					*Actinoplanes rectilineatus*	(68)	
079	52187	52939	27.5 (V)	DNA polymerase III subunit epsilon	WP_026118477	121/234	
				(DnaQ-like exonuclease)	*Nocardiopsis salina*	(52)	
080	52936	53097	5.9 (V)		No match		
081	53097	53723	22.6 (M)		No match		
082	53720	54208	17.9 (V)	Holliday junction resolvase	WP_033299115	102/157	
					*Nocardiopsis gilva*	(65)	
083	54205	54645	16.3 (M)	WhiB family transcriptional regulator	WP_011595908	50/107	
					*Rhodococcus*	(47)	
084	54648	55439	30.3 (M)	Hypothetical protein	WP_017602245	135/271	
					*Nocardiopsis lucentensis*	(50)	
085	55657	55343	11.0 (M)		No match		38
086	55382	56044	24.7 (V)	Hypothetical protein	WP_043984960	80/215	
					*Mycobacterium llatzerense*	(37)	
087	56029	57438	51.9 (V)	Hypothetical protein	EFE65835	135/380	
					*Streptomyces ghanaensis* ATCC 14672	(36)	
088	56919	56311	21.1 (L)		No match		40
089	57435	58103	24.4 (M)	Hypothetical protein	WP_030282501	30/83	63
					*Streptomyces* sp. NRRL B-5680	(36)	
090	58214	58504	14.5 (V)		No match		25
091	58573	58908	12.3 (M)	Hypothetical protein	WP_037865813	61/118	
					*Streptomyces* sp. NRRL S-1868	(52)	
092	58872	58621	9.3 (M)		No match		
093	59867	59571	10.5 (V)		No match		

a *The first translated amino acid is shown in parentheses*.

b *The matched homologs in Streptomyces phage phiSASD1 are also included although they are not the best hits*.

c *The percentage identity is calculated based on the number of identical amino acid residues (numerator) over the number of compared residues (denominator) and shown in parentheses*.

d *The sequence coverage (%) determined by mass spectrometry of the protein is indicated*.

## Results and discussion

### Isolation and purification of phage P1312

**Figure 1:**
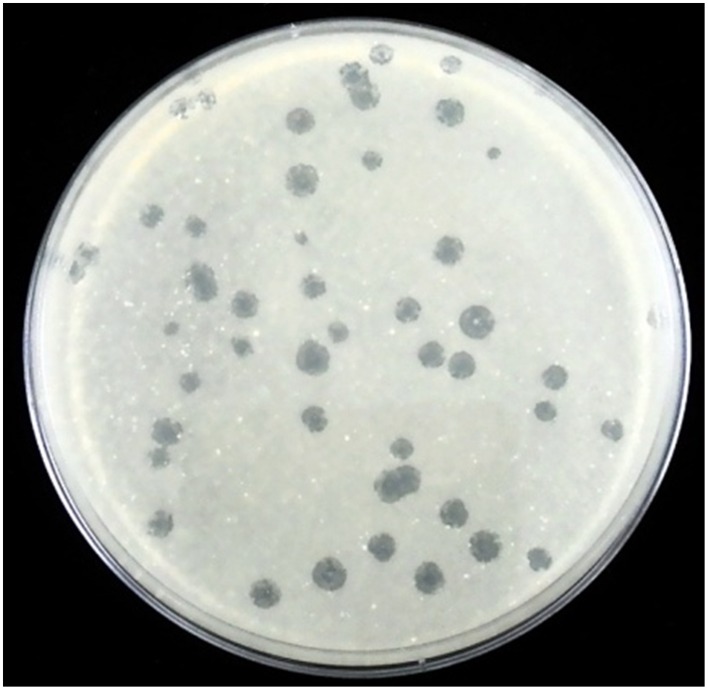
**Plaques of P1312 in the lawn of *T. fusca* NTU22**.

**Figure 2:**
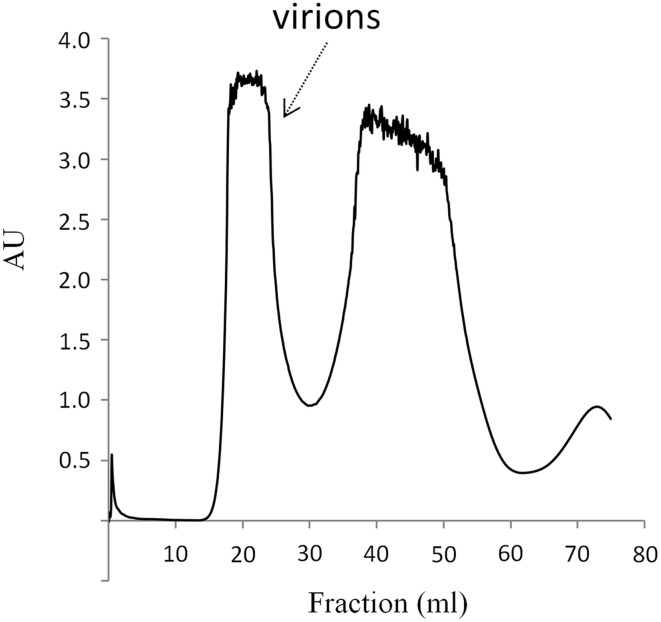
**Purification of P1312 by gel filtration chromatography**. The PEG-precipitated P1312 was loaded into a 50 ml Sephacryl S-500HR column. The proteins were eluted with TBS buffer and monitored with a UV detector. AU is referred to absorbance units.

Compost collected from several suburban farms was tested for the presence of phages that infect *T. fusca* according to the method described in Materials and Methods. A chicken manure sample was found to contain phages that could grow in *T. fusca* and resulted in lytic plaques in the lawn of the bacterium (Figure 1). After plaque purification and phage propagation, the virions were purified by PEG precipitation and gel filtration using a Sephacryl S-500HR column as described in Materials and Methods. The phage particles could be obtained from the fractions corresponding to the void volume of the chromatography (Figure 2). According to the TEM photos and the genome organization (described below), we believe that the purified sample contained only one type of phage. If there were more than two bacteriophages, more genes for phage specific proteins such as tape measurement protein and large subunit of terminase would be expected.

### Infectivity of P1312

**Figure 3:**
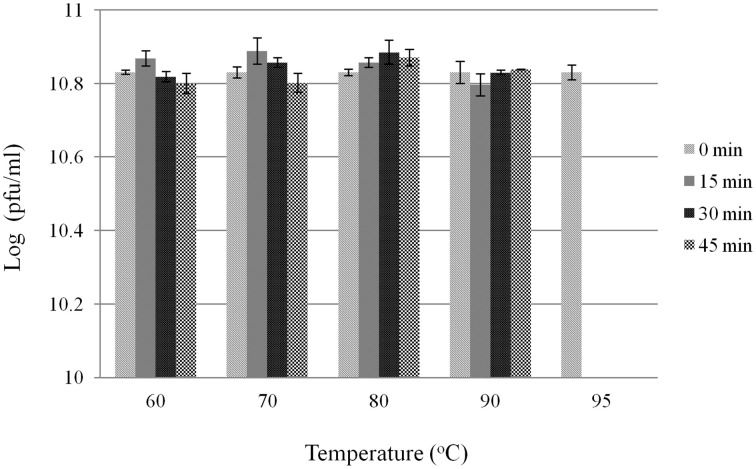
**Thermal stability of *T. fusca* phage P1312**. P1312 diluted in water was incubated at 60, 70, 80, 90, and 95°C for the indicated time, and the residual infectivity was determined by the plaque assay.

**Figure 4:**
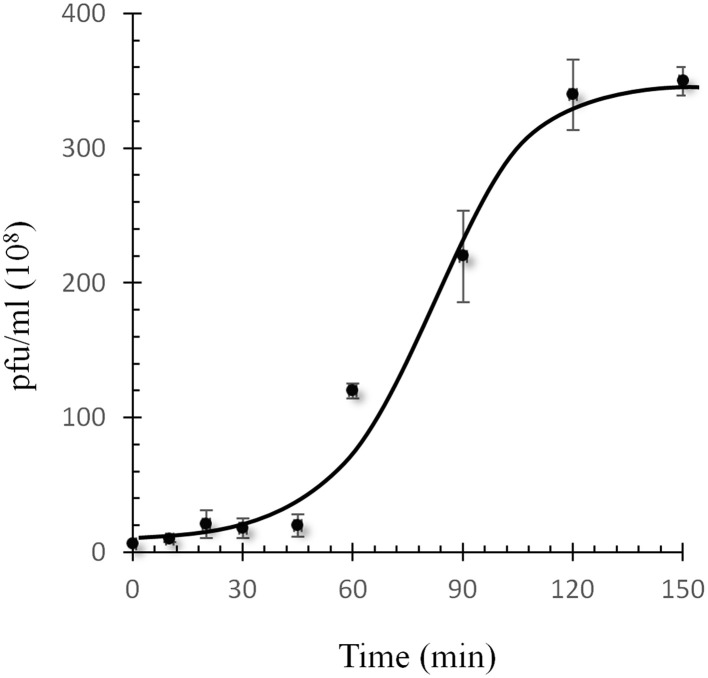
**One-step growth curve of P1312 at 50°C**. Bacterial cultures were infected with a MOI of 1.0. The phage adsorption and culture conditions were as the description in Materials and Methods. Error bars indicate the standard deviation in triplicate samples.

Since *T. fusca* is a moderately thermophile, it was interesting to know the thermal stability of P1312. The phage particles were incubated at 60, 70, 80, 90, and 95°C. Aliquots were withdrawn periodically during the incubation and the residual infectivity was determined by the plaque assay. The infectivity of P1312 remained intact after the incubations at 90°C for 45 min, indicating that P1312 is extremely thermostable (Figure 3). Nonetheless, 5 min incubation at 95°C was able to inactivate P1312 effectively. For one-step growth experiments, P1312 was added into the culture of *T. fusca* at an MOI of 1. After 10 min incubation, which resulted in 69% adsorption of the phage particles to the host, the infected host cells were transferred into a fresh CYC medium and the phage particles released into the medium during the following cultivation were determined by the plaque assay. There was a latent period of ~45 min before the rapid increase of the phage titer, and lysis was complete by ~120 min (Figure 4). The growth kinetics of P1312 suggests that the average burst size is 57 pfu per infected cell.

### General features of P1312

**Figure 5:**
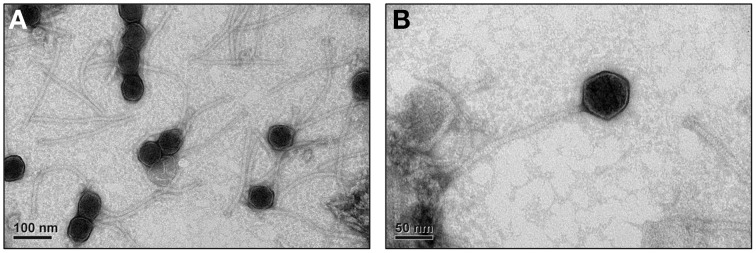
**Electron microgram of P1312 virions**. The phage particles were prepared, negatively stained and examined by electron microscope as described in Materials and Methods. **(A)** The broad view of the phage particles. **(B)** The close-up of a single phage particle.

The morphology of P1312 was visualized using a transmission electron microscope (Figure 5). P1312 has a head in hexagonal outline, plausibly icosahedral, and a long flexible non-contractile tail, suggesting that P1312 belongs to the family *Siphoviridae*. The head is approximately 56 nm in diameter, and the tail is approximately 250 nm long and 11 nm wide. The complete nucleotide sequence of the genome of P1312 was determined with Illumina Miseq and assembled into a circular genome of 60,284 base pairs using the assembly algorithm of CLC Genomics Workbench. The GC content of the phage genome is 65.9%, close to 67.5% of the host's chromosome (Lykidis et al., 2007). The possible ORFs in the genome were predicted by Bacterial Annotation System and Glimmer/RBSfinder program. Integrating the computing results indicated the presence of 93 ORFs, arranged on both strands. Among them, 36 ORFs start with methionine, while 44 and 13 ORFs start with valine and leucine, respectively. The biggest ORF encodes a protein containing 1657 amino acid residues; by contrast, the smallest one encodes a peptide of 30 amino acids. The general information of the proteins encoded by the ORFs regarding their lengths, putative functions, and homologs found by BLASTp are listed in Table 1. The phage head morphogenesis protein-encoding ORF was arbitrarily assigned as the first ORF, followed by terminase genes, so that the presented gene order is similar to that of many bacteriophages such as *Salmonella* bacteriophage ES18 (Casjens et al., 2005) and *Enterococcus faecalis* bacteriophage ϕEf11 (Stevens et al., 2011). Thirty-one ORFs encode proteins that contain acknowledged functional domains or motifs, while 23 ORFs encode hypothetical proteins that have homologs found in other phages, prophages or bacteria, despite the lack of putative conserved domains. The rest of the predicted ORFs encode proteins sharing no significant identity with proteins in the databases; therefore, some of them may represent false positives.

The purified phage particles were subjected to protein identification by LC-MS/MS. This method identified 24 out of the 93 predicted proteins, including many phage structural proteins and enzymes such as N-acetylmuramoyl-L-alanine amidase and replicative DNA helicase (Table 1). Nine identified proteins (encoded by ORF6, 10, 14, 50, 75, 76, 85, 88, and 90) actually do not have significant homologs in the databases; therefore, they may represent novel proteins. It is noteworthy that either DNase I or RNase A, which were used in the pretreatment prior to the size exclusion chromatography, was not detected. The absence of the nucleases in the phage preparation suggests that the chromatographic method could effectively separate the soluble proteins from the virions.

### Provisional functions of P1312 proteins

**Figure 6:**
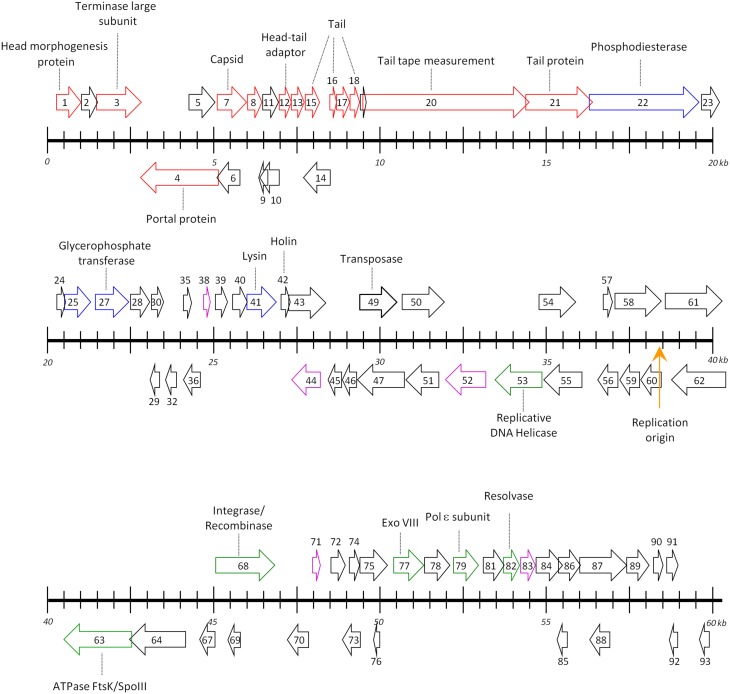
**Map of the genome of P1312**. The apparently circular genome is open arbitrarily at the upstream region of the head morphogenesis protein-encoding gene. Rightward arrowed boxes denote the ORFs encoded by the forward strand, while leftward arrowed boxes represent those encoded by the complementary strand. ORFs colored by red, blue, green, and magenta direct the synthesis of structural proteins, enzymes involved in DNA degradation/modification and cell wall modifications, enzymes participating in phage DNA replication, and transcriptional factors, respectively. The orange arrow denotes the presumed origin of DNA replication. Ticks on the scale are at intervals of 500 bp.

**Figure 7:**
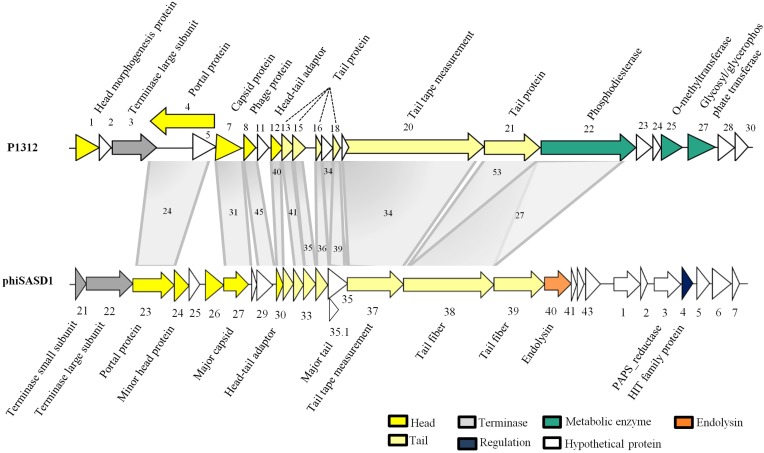
**Comparison of the head and tail gene modules between P1312 and phiSASD1**. The percentage identity between the compared proteins (see details in Table 1) is shown within the gray shadow.

Genome organization of P1312 is illustrated in Figure 6. ORFs are displayed on both DNA strands with many of them overlapping. The cluster encompassing ORF1–21 is responsible for phage particle formation. ORF1 produces the head morphogenesis protein. The product of ORF3 contains an ATP-binding cassette transporter nucleotide-binding domain and shares similarity to the large subunit of phage terminases. ORF4 is supposed to produce the phage portal protein, the junction between the phage head and tail proteins, through which DNA passes during packaging and injection. ORF7 encodes a phage capsid protein. The product of ORF8 belongs to a family composed of proteins from a variety of bacteriophages. The region from ORF12 to ORF21 probably is involved in tail formation. In details, ORF12 directs the synthesis of the phage head-tail adaptor, while ORF13, 15, 16, 17, 18, and 21 likely encode the phage tail components. ORF20, the largest gene in the genome, encodes the tail tape measure protein. For lambdoid phages, the size of the tape measurement protein corresponds to the tail length by a fairly constant of 0.15 nm per amino acid residues (Katsura, 1990). The average tail length of P1312 is approximately 250 nm, accordant to the size of the phage tail tape measure protein. Many of the proteins encoded by the assumed head and tail gene modules share similarities to the proteins encoded by *Streptomyces* phage phiSASD1 (Wang et al., 2010) (Table 1). The comparison of the modules between P1312 and phiSASD1 is shown in Figure 7. Despite the resemblance, a couple of differences in the gene order are noticed. First, the directions of portal protein gene are opposite. Second, the N terminus of gp38 of phiSASD1 shares a small similar region with a tail protein of P1312 (product of ORF21), while the C terminus has a region similar to the CBM_4_9 domain present in the phosphodiesterase of P1312 (product of ORF22). Apart from the head and tail gene modules, the other modules share less similarity between P1312 and phiSASD1.

The segment from ORF22 to ORF49 is notable for producing enzymes for a variety of functions such as DNA degradation/modification and cell wall disruption. ORF22 encodes a protein of 1096 amino acid residues. This protein is predicted to contain an N-terminal signal peptide, a metallophosphatase (MPP) domain and a domain related to carbohydrate-binding module (CBM) family 4_9 at the C-terminus. Proteins in the MPP superfamily are functionally diverse; the members include Mre11/SbcD-like exonucleases, Dbr1-like RNA lariat debranching enzymes, YfcE-like phosphodiesterases, purple acid phosphatases (PAPs), YbbF-like UDP-2,3-diacylglucosamine hydrolases and acid sphingomyelinases according to the conserved domain database, NCBI. By the N-terminal signal peptide and the C-terminal CBM, the product of ORF22 may be secreted through the cell membrane and anchors within the cell wall architecture. The MPP domain is thought to have a DNA degradation activity that may prevent the superinfection of the host by other bacteriophages. The cell wall of *T. fusca* mainly contains peptidoglycan and polyglycerolphosphate-lipoteichoic acid (Rahman et al., 2009); the latter is covalently linked to the outer leaflet of the cytoplasmic membrane. Therefore, the alternative function of the MPP domain may be involved in the hydrolysis of the cell wall by hydrolyzing the phosphodiester bonds in the teichoic acid polymers. ORF25 directs the synthesis of a putative class I adenosylmethionine-dependent methyltransferase. Presumably, this enzyme has an activity for nucleic acid modification. ORF27 encodes a protein similar in amino acid sequence to glycosyl/glycerophosphate transferase, a protein responsible for the polymerization of the main chain of the cell wall-associated teichoic acid. The product of ORF27, as being a phage protein, is thus assumed to interfere with, rather than promote, the host's function of teichoic acid synthesis.

The protein encoded by ORF41 is a putative N-acetylmuramoyl-L-alanine amidase, presumably able to disintegrate the peptidoglycan of the host cell by cleaving the amide bond between N-acetylmuramoyl and L-alanine. It is noteworthy that this phage protein does not contain a secretory signal sequence, consistent with a long-term observation that the endolysin produced by double stranded DNA bacteriophages requires a small membrane protein, known as a holin, to permeabilize the membrane for its access to the peptidoglycan (Young et al., 2000). Interestingly, we found that this putative amidase was associated with the phage particles according to the results of tandem mass spectrometry. This association may reflect an artifact caused by an unspecific interaction of the protein to the virion. Alternatively, it may implicate an involvement of the amidase in the infection step by assisting the phage to inject its DNA into the host cell. Actually, tail-associated peptidoglycan-degrading enzymes involved in localized cell wall degradation have been evidenced in a number of bacteriophages such as Tuc2009 (Kenny et al., 2004) and ϕ29 (Xiang et al., 2008). ORF042 encodes a 93-amino acid polypeptide with two transmembrane helices predicted by the TMPred program (Hofmann and Stoffel, 1993). It is tempting to assume that this polypeptide serves as the holin for the phage amidase to pass through the membrane. ORF49 encodes a transposase belonging to IS605 family, which is also present in the genome of *T. fusca* and many other bacteria. Presumably, phage P1312 acquired this gene from its bacterial host.

Genes responsible for DNA replication and transcriptional regulation are situated sporadically in the remaining half of the genome. Presumably, ORF53 directs the synthesis of a DnaB-like replicative helicase that unwinds the DNA duplex at the replication fork. ORF63 encodes a protein whose C-terminus shares similarity to DNA translocase ftsK of *Actinoplanes*, suggesting the involvement of the protein in DNA segregation. The protein encoded by ORF68 contains a putative serine recombinase domain that is usually found associated with pfam00239 in putative integrases/recombinases of mobile genetic elements of diverse bacteria and phages. Therefore, this protein may catalyze the integration and excision of the phage genome in and from the host chromosome, respectively. It is noteworthy that P1312 behaved as a lytic bacteriophage in this study; therefore, the culture condition may disfavor the integration function of the protein probably through suppressing the protein expression. The protein encoded by ORF77 is related to the PDDEXK superfamily, and appears to be an exonuclease VIII. The product of ORF79 is a DnaQ-like exonuclease, belonging to DEDDh 3′-5′ exonuclease family. Probably, it acts as a proofreading subunit (epsilon) of polymerase III for the DNA replication of P1312. ORF 82 encodes a protein of RusA superfamily that can resolve Holliday junction intermediates by its endonuclease activity. BLASTp analysis also suggests the presence of five transcriptional regulators, encoded by ORF38, 44, 52, 71, and 83. P1312 may use them to control the gene expressions, in terms of transcriptional timing and level, for its successful proliferation. The probable replication origin of P1312 was predicted by the GenSkew program, an application for computing and plotting nucleotide skew data. The resulting GC-skew plot (not shown) suggests that the region around the nucleotide 38440 (close to ORF60) could be the initiation site for replication.

### DNA packaging strategy

**Figure 8:**
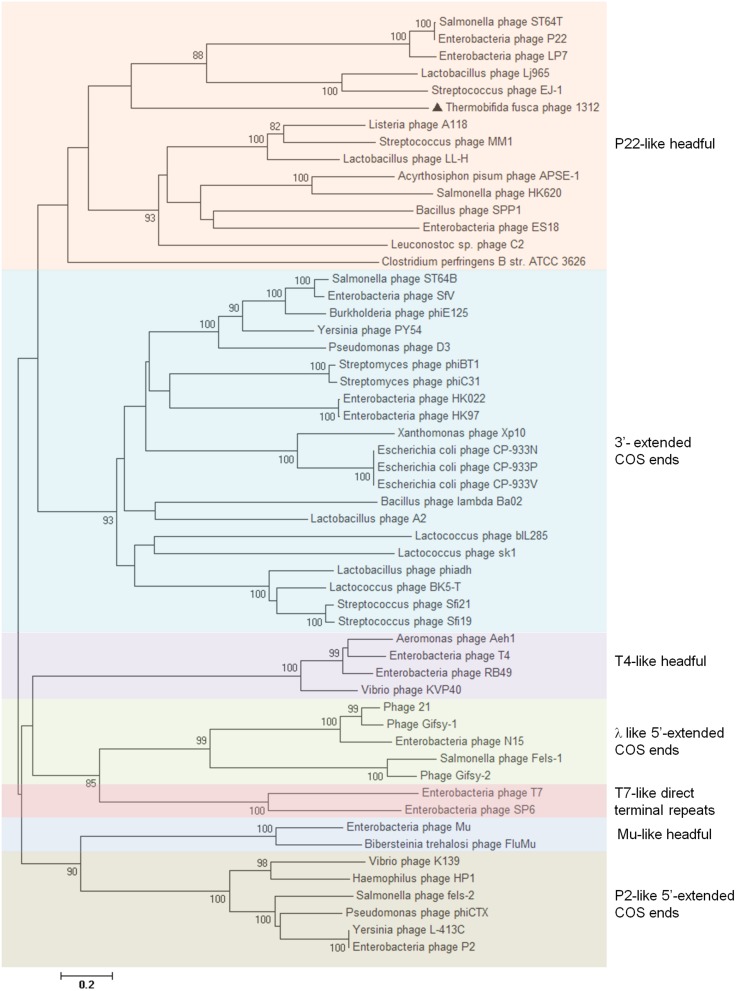
**Neighbor-joining phylogenetic tree of large terminase subunit amino acid sequences**. The sequences, except that from P1312, were classified as described previously (Casjens et al., 2005), and bootstrap analysis was performed with 1000 repetitions. The node of phylogenetic tree shows the bootstrap confidence values above 50%.

**Figure 9:**
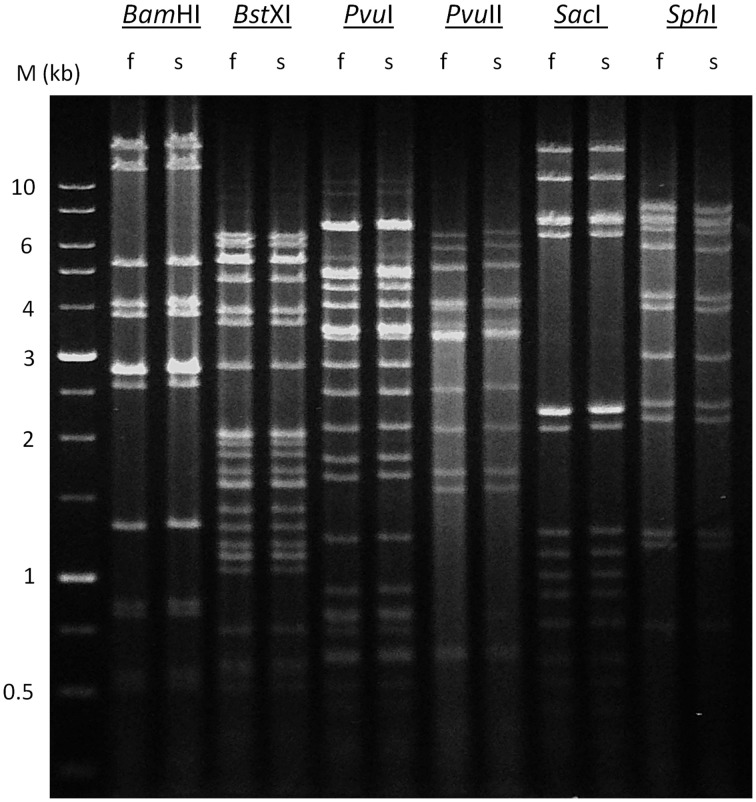
**Restriction analysis of P1312 DNA**. The phage DNA was completely digested with *Bam*HI, *Bst*XI, *Pvu*I, *Pvu*II, *Sac*I or *Sph*I, and the products were analyzed by 0.8% agarose gel electrophoresis. Lane M indicate DNA marker, 1-kb DNA Ladder. f and s indicate that the digests were heated to 80°C for 15 min and then cooled fast or slow to room temperature, respectively.

All known tailed-bacteriophage virions contain a single linear dsDNA chromosome, because the passage of the portal protein is not wide enough to allow two parallel dsDNAs to be threaded simultaneously into the head during packaging and out of the virion during injection. As a member of *Siphoviridae*, P1312 probably contains a linear genome, rather than a circular one as determined by whole genome sequencing using Illumina Miseq. Six types of termini of the chromosomes in tailed-bacteriophage virions have been studied (Casjens and Gilcrease, 2009); they are (1) single-stranded cohesive ends, (2) circularly permuted direct terminal repeats, (3) short, non-permuted, direct terminal repeats, (4) long, non-permuted, direct terminal repeats, (5) terminal host DNA sequences, and (6) covalently bound terminal proteins. These different types of ends reflect varied DNA replication strategies and depend on terminase actions during DNA packaging. A previous bioinformatic analysis indicated that the different functional classes of phage-encoded terminases can usually be predicted from the amino acid sequence of large terminase subunits (Casjens et al., 2005). According to the determined nucleotide sequence, the termini of type 5 and 6 were precluded from the genome of P1312. To have a clue about the most probable packaging strategy employed by P1312, the large terminase subunit of P1312 was compared, in amino acid sequence, to those of bacteriophages with known chromosomal termini. Based on the resulting phylogenetic tree (Figure 8), P1312 is classified into a clan that uses the P22-like headful strategy for DNA packaging. Accordingly, the genomic termini of P1312 are possibly circularly permuted direct terminal repeats. To support this proposition, the phage genome was digested with a variety of restriction enzymes, and the digested products were heated at 80°C for 15 min, followed by a fast or slow cooling process. The samples were then analyzed by agarose gel electrophoresis (Figure 9). If the genome has single-stranded cohesive ends, the two restriction fragments, which have a single cohesive terminus, will join together and appear as a larger fragment in the slow chilled sample. That the restriction patterns of P1312 did not alter in response to the different cooling processes excludes the possibility of the presence of cohesive ends. For headful packaging phages that contain terminally redundant and circularly permutated chromosomes, the restriction pattern may consist of all the fragments expected from a circular genome plus a submolar pac fragment as in the case of P22 (Casjens and Gilcrease, 2009). However, if the headful terminase makes imprecise series initiation cleavage, as is the case for sf6 and ES18, no visible pac fragment will be detected in the restriction pattern. Instead, the terminal fragments will show as blur backgrounds between bands due to their variable lengths (Casjens and Gilcrease, 2009). The restriction patterns created by the various restriction enzymes in Figure 9 are consistent with the predicted results based on a circular P1312 genome. No pac fragment was observed in the electrophoresis gel in this study; however, blur backgrounds were actually present. This result supports the proposition that P1312 is a P22-like headful packaging phage.

## Concluding remarks

Bacteriophage P1312 is the first well-described phage to infect *T. fusca*. The tolerance to high temperature indicates that P1312 has adapted to the thermophilic stage of composting as *T. fusca*. In general, P1312 has a genome organization similar to *Siphoviridae* of Gram-positive bacteria; however, a notable difference is the direction of the portal protein-encoding gene, which renders the genome of P1312 unique. *Nocardiopsis lucentensis*, an actinomycete lives in saline soil habitats (Yassin et al., 1993), is the number 1 ranked organism that provides best-matched homologs with the proteins encoded by the putative ORFs of P1312. This fact suggests the presence of prophages in *N. lucentensis* or implies a possibly evolutionary origin of P1312. P1312 may represent a valuable resource for understanding the molecular interaction between *T. fusca* and its co-evolutionary bacteriophages.

### Conflict of interest statement

The authors declare that the research was conducted in the absence of any commercial or financial relationships that could be construed as a potential conflict of interest.
